# Clinical outcomes and hemorrhagic or thromboembolic risks in decompressive craniectomy for patients taking antiplatelet or anticoagulant therapy

**DOI:** 10.1007/s10143-025-03491-4

**Published:** 2025-03-26

**Authors:** Alba Scerrati, Giovanni Scanferla, Lorenzo Sgarbanti, Giorgio Mantovani, Chiara Angelini, Maria Elena Flacco, Rosario Maugeri, Lapo Bonosi, Domenico Gerardo Iacopino, Silvana Tumbiolo, Alessandro Adorno, Lara Brunasso, Giorgio Lofrese, Vittoria Rosetti, Luigino Tosatto, Teresa Somma, Luigi Maria Cavallo, Sara Lombardi, Carmelo Lucio Sturiale, Francesco Signorelli, Anna Maria Auricchio, Grazia Menna, Luca Ricciardi, Nicola Montemurro, Fabio Raneri, Oriela Rustemi, Giampaolo Zambon, Michele Alessandro Cavallo, Pasquale De Bonis

**Affiliations:** 1 Department of Translational Medicine, University of Ferrara, Ferrara, Italy; 2 Department of Neurosurgery, Sant’Anna University Hospital of Ferrara, Ferrara, Italy; 3 Environmental and Preventive Sciences, University of Ferrara, Ferrara, Italy; 4 Unit of Neurosurgery, AOUP “Paolo Giaccone”, Department of Biomedicine Neurosciences and Advanced Diagnostic (BiND), University of Palermo, Palermo, Italy; 5 Unit of Neurosurgery Hospital “Villa Sofia”, Palermo, Italy; 6 Neurosurgery Unit, Bufalini Hospital, Cesena, Italy; 7 Department of Neurosciences and Reproductive and Dental Sciences, Division of Neurosurgery, Federico II University of Naples, 80131 Naples, Italy; 8 Department of Neurosurgery, Institute of Neurosurgery, Fondazione Policlinico Universitario A. Gemelli IRCCS, Università Cattolica del Sacro Cuore, L.Go A. Gemelli 8, 00168 Rome, Italy; 9 Neurosurgical Unit, Department of Neuroscience, Mental Health, and Sensory Organs, Sapienza University of Rome, Rome, Italy; 10 Department of Neurosurgery, Azienda Ospedaliero Universitaria Pisana, University of Pisa, Pisa, Italy; 11 Department of Neurosurgery, ULSS8 Berica, Vicenza, Italy

**Keywords:** Decompressive craniectomy, Anticoagulant, Antiplatelet, Hemorrhagic complications, Thromboembolic complications, CHA₂DS₂-VASc

## Abstract

Decompressive craniectomy (DC) is a critical surgical intervention for elevated intracranial pressure. However, the impact of preoperative antiplatelet or anticoagulant therapy on outcomes and complications remains unclear. A retrospective-prospective study was conducted on 145 patients undergoing DC between November 2021 and May 2023. Patients were categorized into two groups: those with (*n* = 48) and without (*n* = 97) preoperative antithrombotic therapy. Demographic data, comorbidities, antithrombotic therapy type and duration, clinical outcomes, and pre-operative risk factors (CHA2DS2-VASc and HAS-BLED scores) were analyzed. While there was a trend towards higher hemorrhagic complications in the antithrombotic therapy group (20.0% vs. 11.3%), this difference was not statistically significant. However, thromboembolic events, primarily stroke (27.7% vs. 9.3%) and acute myocardial infarction (10.6% vs. 0.0%), were significantly more frequent in the antithrombotic therapy group. Multivariate analysis revealed that ischemic stroke as a primary diagnosis, rather than antithrombotic therapy itself, was a significant predictor of thromboembolic complications (adjusted OR 3.49, 95%CI 1.47–8.28, *p* = 0.005). Pre-operative GCS was associated with improved outcomes (adjusted OR 0.81, 95%CI 0.67–0.97, *p* = 0.025). While antithrombotic therapy does not appear to increase the risk of hemorrhagic complications after DC, it is associated with a higher risk of thromboembolic events, especially in patients with ischemic stroke. Individualized assessment and tailored management of antithrombotic therapy are crucial to optimize outcomes in DC patients. Further studies are needed to refine strategies for bridging anticoagulation and managing antithrombotic therapy in this population, considering factors such as CHA2DS2-VASc and HAS-BLED scores, as well as patient-specific risk profiles.

## Introduction

Decompressive craniectomy (DC) is an emergency surgical procedure indicated for the immediate reduction of intracranial pressure (ICP) in acute conditions such as major head trauma with brain swelling or contusions, acute subdural hematoma [1–3], and intraparenchymal [4] or subarachnoid bleeding.

However, less is known about the specific risks associated with the prior use of antiplatelet and anticoagulant drugs. In fact, it remains unclear whether the use of these medications should definitively discourage the procedure due to the potentially high rates of re-bleeding and mortality. The impact of preoperative administration of antiplatelet and anticoagulant medications on outcomes and complications in patients undergoing DC is not well understood, and few studies focused on this topic [5–8]. These drugs are widely used among the elderly for cardiovascular and cerebrovascular diseases, but data about the perioperative neurosurgical management of anticoagulant and antiplatelet therapy are limited [9, 10].

Scores such as CHA₂DS₂-VASc and HAS-BLED are used to estimate the risk of thromboembolic and bleeding events in patients with non-valvular atrial fibrillation, thereby guiding therapeutic decisions. These scores have proven to be effective in predicting complication risks during the perioperative period of non-cardiac surgeries [11–13]; however, their role and application in the neurosurgical field remain unexplored [5].

Indeed, neurosurgical patients face a high risk of thromboembolic events like deep vein thrombosis, pulmonary embolism, and stroke. This risk is due to multiple factors, including immobility during and after surgery, use of vasopressors, dehydration, motor deficits, and a hypercoagulable state caused by trauma, malignant tumors or subarachnoid hemorrhage [14]. Moreover, if they are already taking anticoagulants or antiplatelets drugs, we could expect this risk is even higher.

The aim of this study is to evaluate the potential influence of anticoagulant and antiplatelet drugs on outcomes and hemorrhagic or thromboembolic complications in patients undergoing decompressive craniectomy. Our hypothesis is that these drugs do not influence the risk of haemorrhagic events but may increase the risk of thromboembolic complications. Additionally, the study will analyze predictive factors such as platelet count, INR values, CHA₂DS₂-VASc and HAS-BLED scores and patient performance status. We will compare our data with the few findings on this topic in the literature [5, 7] showing no influence of antiplatelets or anticoagulants on the risk for hemorrhagic events. About the management of these drugs and the thromboembolic risk in patients undergoing DC, no published clinical studies are available.

## Materials and methods

A multicenter prospective observational study was conducted. All patients who underwent decompressive craniectomy between November 2021 and May 2023 were enrolled.

Data were collected anonymously. The study was approved by the local ethics committee (Prot. 842/2023 I.5/1).

We collected the following data: age, sex, diagnosis leading to surgery (traumatic brain injury (TBI) without acute subdural hematoma (ASDH), TBI with ASDH, intracerebral hemorrhage, and ischemic stroke; additional diagnoses, whether they were the cause of the procedure or complementary to one of the main indications were collected separately in a single category), intracranial pressure (ICP), use of anticoagulants or antiplatelets both preoperatively (acetylsalicylic acid (ASA), others antiplatelets, Vitamin K antagonists (VKA), Novel Oral AntiCoagulants (NOAC), heparin, antiplatelet + anticoagulant, Dual AntiPlatlet Therapy (DAPT)) and postoperatively (heparin for DVT prophylaxis or ASA) and their management, thromboembolic and hemorrhagic risk factors, CHA₂DS₂-VASc and HAS-BLED scores, comorbidities and Charlson Comorbidities Index (CCI), tobacco and alcohol abuse, postoperative hemorrhagic and thromboembolic events, length of stay in the intensive care unit, pre and post operative Modified Rankin Scale (mRS) and Glasgow Coma Scale (GCS), pre and post operative laboratory exams, 1 and 6 months post operative Glasgow Outcome Scale (GOS).

In patients treated with antithrombotic therapy before surgery, we recorded whether they discontinued the drugs, the duration between suspension and surgery, and the time from surgery to the resumption of therapy. Additionally, perioperative treatment with anticoagulant antagonists, such as platelets, fresh frozen plasma, and vitamin K, was evaluated.

Post-operative radiological follow-up with CT scans was conducted to identify any complications. For hemorrhagic complications (bleedings in the surgical field), events occurring within 30 days after surgery were considered. We documented the time elapsed between surgery and the complication, and, in cases where antithrombotic therapy was resumed, the time between the hemorrhagic event and the resumption of therapy. Thromboembolic complications were categorized into three main types: stroke, myocardial infarction, and others. Additionally, the time elapsed from the discontinuation of antithrombotic therapy to the onset of the thromboembolic event was documented. The patient's coagulation status was analyzed preoperatively and immediately postoperatively using INR.

### Statistical analysis

Standard univariate analyses were used to assess differences in the selected demographic and clinical variables, by therapy status (anti-coagulant/anti-platelet therapy assumption versus no therapy). Chi-squared test was used for categorical variables; t-test and Kruskal–Wallis test were used for parametric and non-parametric continuous variables, respectively (distribution assessed through Shapiro–Wilk test). Multivariate logistic regression was also carried out to evaluate the association between anticoagulant-anti-PLT therapy status and (a) post-surgical onset of hemorrhagic events; (b) post-surgical onset of thrombotic events (acute myocardial infarction, stroke, other minor events), and (b) unfavorable GOS, both one and six months after surgery (total score <4), adjusting for selected potential confounders.

Covariates were included using a stepwise forward process, with therapy (yes vs. no) and age (5-year increase) forced to entry. A covariate/outcome ratio of 1/10 was kept in all phases of model building to avoid overfitting and, given the limited number of successes, the following criteria were strictly adopted to select the other covariates: (a) clinical relevance; (b) in case of multicollinearity (as for HAS-BLED and CHA2DS2-VASc scores—Spearman rho 0.80), the covariate with the higher R 2 was included in the final model. Statistical significance was set at *p* < 0.05, and all analyses were carried out using Stata software, version 13.1 (Stata corp., TX, US, 2014).

## Results

### Demographic and diagnosis at admission

The study enrolled 145 patients, 48 were assigned to the anticoagulant/antiplatelet therapy (AAPT) group and 97 to the no-therapy (NT) group. The mean age was 55.3 years, with a statistically significant higher value in patients in the AAPT group (64.2 vs. 51.0, *p* < 0.0001). No differences were observed for the sex between the 2 groups (Table 1).

**Table 1:** Demography diagnosis at admission of the sample, overall and by therapy status (anticoagulant/antiplatelet—anti-PLT—therapy versus no therapy)

	Overall sample	Anticoagulant- Anti-PLT therapy	No therapy	
Variables	(*N* = 145)	(*N* = 48)	(*N* = 97)	p *
Mean age in years (SD)	55.3 (16.7)	64.2 (13.3)	51.0 (16.6)	** < 0.001**
Male gender, %	42.1	41.7	42.3	0.9
Trauma without aSDH, %	7.6	8.3	7.2	0.8
Trauma with aSDH, %	44.8	39.6	47.4	0.4
*Diagnosis at baseline:*				
Intracerebral haemorrhage, %	39.3	35.4	41.2	0.5
Stroke, %	25.5	37.5	19.6	**0.02**
Others, %	35.9	27.1	40.2	0.12
	(*N* = 70)	(*N* = 16)	(*N* = 54)	
Median post-operative ICP in mmHg (IQR)	13.0 (8.0–18.0)	10.0 (7.0–14.0)	15.0 (8.0–23.0)	0.06

^*^ Chi-squared test for categorical variables; t-test and Kruskal–Wallis test for parametric and non-parametric continuous variables, respectively

*SD* standard deviation, *IQR* interqurtile range, *aSDH* acute subdural hematoma, *ICP* intracranial pressure, *DVT* deep vein thrombosis, *CCI* Charlson Comorbidity Index, *GCS* Glasgow Coma Scale

^A^ Warfarin, Coumarins; ^B^ Rivaroxaban, Dabigatran, Apixaban, ecc

Most of the patients in the overall population (44.8%) suffered from a trauma with acute subdural hematoma (aSDH—39.6% and 47.4% respectively in the AAPT and NT group, *p* = ns).

More than one-third of the patients suffered from intracerebral hemorrhage (39.3%), and a quarter of the patients from an ischemic stroke (25.5%). About 36% of patients had also other diagnoses such as brain contusions, subarachnoid hemorrhage, etc. Only for stroke there was a statistically significant difference: 37.5% vs. 19.6% in the AAPT and NT group respectively; *p* = 0.020.

### Baseline anticoagulant/anti-PLT therapy in the AAPT group

A total of 48 patients were taking anticoagulants or antiplatelets preoperatively. Acetylsalicylic acid (ASA) was the most common used drug (54.2%).

Drug discontinuation was reported in 77.1% of patients with a median time between drug discontinuation and surgery of 1 day [min = 0; max = 4].

About one-third (31.0%) of patients in the overall population (27.1% and 33.0%, *p* = ns, in the AAPT and NT group respectively) underwent postoperative prophylaxis with heparin for deep venous thrombosis (DVT) prevention. Four patients (8%) restarted ASA with a median time between surgery and drug resumption of 2 days (min = 0; max = 5 days).

No significant differences were found in terms of peri-intraoperative antagonist transfusion between the 2 groups, with platelets as the most common antagonist transfused (Table 2).

**Table 2:** Baseline anticoagulant/anti-PLT therapy in the AAPT group

	Overall sample	Anticoagulant- Anti-PLT therapy	No therapy	
Variables	(*N* = 145)	(*N* = 48)	(*N* = 97)	p *
*Baseline anticoagulant/anti-PLT therapy:*				
Acetylsalicylic acid, %	17.9	54.2	–	–
Antiplatelets, %	5.5	16.7	–	–
Vitamin-K antagonist ^A^, %	3.5	10.4	–	–
Non-VKA Oral Anticoagulants (NOACs), % ^B^	4.1	12.5	–	–
Antiplatelet + Anticoagulant, %	4.8	12.5	–	–
Dual antiplatelet, %	4.1	10.4	–	–
*Drug management:*				
Drug discontinuation, %	27.1	77.1	–	–
Median time between drug discontinuation and surgery, in days (IQR)	–	1.0 (0.0–1.0)	–	–
Median time between surgery and drug resumption, in days (IQR)	–	2.0 (0.0–5.0)	–	–
*Peri-intraoperative antagonists transfusion:*				
Platelet, %	9.7	12.5	8.3	0.4
Fresh frozen plasma, %	6.2	10.4	4.1	0.14
Vitamin K, %	4.1	8.3	2.1	0.07
*Post-surgical drugs administration:*				
Heparin for DVT prevention, %	31.0	27.1	33.0	0.5
Acetilsalycilic acid, %	6.2	8.0	3.1	0.7

^*^ Chi-squared test for categorical variables; t-test and Kruskal–Wallis test for parametric and non-parametric continuous variables, respectively

*SD* standard deviation, *IQR* interqurtile range, *aSDH* acute subdural hematoma, *ICP* intracranial pressure, *DVT* deep vein thrombosis, *CCI* Charlson Comorbidity Index, *GCS* Glasgow Coma Scale

### Pre-operative risk and clinical scores

Median CHA_2_DS_2_-Vasc, HAS-BLED and CCI scores showed statistically significant differences between the 2 groups (*p* < 0.001) with higher scores in the AAPT group (Table 3).

**Table 3:** Pre-operative risk and clinical scores

	Overall sample	Anticoagulant- Anti-PLT therapy	No therapy	
Variables	(*N* = 145)	(*N* = 48)	(*N* = 97)	p *
Median CHA2DS2-VASc score (IQR)	2.0 (0.0–4.0)	4.0 (3.0–5.0)	1.0 (0.0–2.0)	** < 0.001**
Median HAS-BLED score (IQR)	1.0 (0.0–3.0)	3.0 (2.0–4.0)	1.0 (0.0–1.0)	** < 0.001**
Median CCI (IQR)	2.0 (1.0–4.0)	4.0 (2.0–6.0)	2.0 (0.0–3.0)	** < 0.001**
*Pre-surgical mRS:*	(*N* = 131)	(*N* = 40)	(*N* = 91)	
Median score (IQR)	4.0 (4.0–5.0)	4.0 (3.0–5.0)	5.0 (4.0–5.0)	0.2
Score categories, %				0.3
0–2—minor disability	16.8	17.5	16.5	
3–4—moderate disability	33.6	42.5	29.7	
5—severe disability	49.6	40.0	53.8	
6—death	0.0	0.0	0.0	
*Pre-surgical GCS:*				
Median score (IQR)	6.0 (4.0–10.0)	6.0 (4.0–10.0)	7.0 (4.0–9.0)	0.9
Score categories, %				0.2
3–8—unresponsive	70.2	64.6	73.1	
9–12—comatose	20.6	29.2	16.1	
13–15 best score	9.2	6.2	10.8	
*Coagulation parameters*				
Preoperative platelets (median (IQR))	212.0(164.0, 269.3)	203.0(155.5, 280.7)	212.0(164.3, 268.5)	0.9
Preoperative INR (median (IQR))	1.09 (1.04, 1.18)	1.11 (1.04, 1.21)	1.09 (1.04, 1.15)	0.5
Postoperative platelets (median (IQR))	202.0 (144.0, 270.0)	211.0(152.0, 279.0)	202.0(141.3, 264.8)	0.6
Postoperative INR (median (IQR))	1.11 (1.05, 1.20)	1.12 (1.06, 1.22)	1.10 (1.04, 1.19)	0.3

^*^ Chi-squared test for categorical variables; t-test and Kruskal–Wallis test for parametric and non-parametric continuous variables, respectively

*SD* standard deviation, *IQR* interqurtile range, *CCI* Charlson Comorbidity Index, *GCS* Glasgow Coma Scale, *GOS* Glasgow Outcome Scale

Considering the pre-operative mRS and GCS categories, most of the patients presented a score of mRS = 5 (severe disability) (49.6%) and GCS = 3–8 (70.2%), with no statistically significant differences between the groups (Table 3).

No statistically significant differences were found between pre- and post-operative platelets count and INR values between the 2 groups.

### Post-operative complications and clinical outcome

Post operative acute hemorrhage in the surgical field (24 h) was reported in 14.1% of patients (20.0 vs 11.3 in the AAPT and NT group respectively, *p* = ns).

New ischemic stroke (not present before treating the patient, and not the cause of the surgery) was reported in 15.3% of patients with a statistically significant difference between the 2 groups (27.7% vs 9.3% in the AAPT and NT group respectively, *p* < 0.001). Higher incidence of acute myocardial infarction was also significant in the AAPT group (10.6 vs 0.0, *p* = 0.001).

Median length of stay in the ICU did not show significant differences with a median of 15.0 days (Table 4).

**Table 4:** Post-operative complications and clinical outcome

	Overall sample	Anticoagulant- Anti-PLT therapy	No therapy	
Variables	(*N* = 145)	(*N* = 48)	(*N* = 97)	p *
*Post-surgical outcomes:*				
Haemorrhage, %	14.1	20.0	11.3	0.2
Stroke, %	15.3	27.7	9.3	** < 0.001**
Acute myocardial infarction, %	3.5	10.6	0.0	**0.001**
Other events, %	13.9	19.2	11.3	0.2
Median post-surgical length of stay in ICU in days (IQR)	15.0 (7.0–27.0)	14.0 (9.0–21.0)	18.0 (6.0–30.0)	0.2
*Post-surgical mRS:*	(*N* = 131)	(*N* = 40)	(*N* = 91)	
Median score (IQR)	4.0 (3.0–5.0)	5.0 (4.0–5.0)	4.0 (3.0–5.0)	0.2
Score categories, %				0.2
0–2—minor disability	8.4	2.5	11.0	
3–4—moderate disability	45.0	45.0	45.0	
5—severe disability	46.6	52.5	44.0	
6—death	0.0	0.0	0.0	
*Post-surgical GCS:*				
Median score (IQR)	10.0 (5.0–12.0)	7.5 (3.0–12.0)	10.0 (5.0–12.0)	0.08
Score categories, %				0.6
3–8—unresponsive	46.7	52.2	44.0	
9–12—comatose	31.4	30.4	31.9	
13–15 best score	21.9	17.4	24.1	
*Post-surgical GOS:*				
1-month median score (IQR)	3.0 (2.0–3.0)	2.5 (1.0–3.0)	3.0 (2.0–3.0)	0.06
1-month score categories, %				**0.01**
1- death	24.5	30.4	21.5	
2–3—severe disability	64.0	69.6	61.3	
4–5—minor disability	11.5	0.0	17.2	
6-month median score (IQR)	3.0 (1.0–3.0)	3.0 (1.0–3.0)	3.0 (2.0–4.0)	**0.046**
6-month score categories, %				**0.021**
1- death	25.6	34.2	21.5	
2–3—severe disability	51.3	57.9	48.1	
4–5—minor disability	23.1	7.9	30.4	

^*^ Chi-squared test for categorical variables; t-test and Kruskal–Wallis test for parametric and non-parametric continuous variables, respectively

*SD* standard deviation, *IQR* interqurtile range, *CCI* Charlson Comorbidity Index, *GCS* Glasgow Coma Scale, *GOS* Glasgow Outcome Scale

Post operative clinical scores were available for 131 patients, 40 and 91 in the AAPT and NT group respectively.

No differences were found between the 2 groups for post-operative mRS and GCS with most of the patients (91.6%) reported as severe or moderate disability or unresponsive-comatose (78.1%) (Table 4).

One month – post operative GOS categories did show statistically significant differences (*p* = 0.01) between the 2 groups, with higher risk for death (1) in the AAPT group (30.4% vs 21.5% in AAPT and NT group respectively and for minor disability (4–5) in the NT group (0.0% vs 17.2% in AAPT and NT group respectively). Similar results were found for the 6-month – post operative GOS (Table 4).

### Univariate and multivariate analysis for risk of post-operative hemorrhages

Univariate analysis and multiple logistic regression was performed to analyze the correlation between the preoperative use of antithrombotics or anticoagulants and the development of postoperative hemorrhage (Table 5).

**Table 5:** Univariate and multivariate analyses evaluating the association between anticoagulant-anti-PLT therapy status and post-surgical onset of hemorrhagic events, adjusting for selected potential confounders

	Post-surgical hemorrhage (*n* = 20)			
	Raw OR (95% CI)	p	Adj. OR (95% CI)	p
*Antithrombotic therapy:*				
No	1 (ref. cat.)		1 (ref. cat.)	
Yes	1.95 (0.75–5.12)	0.2	2.13 (0.79–5.72)	0.13
*Age:*				
5-year increase	1.06 (0.90–1.23)	0.4	–	–
*Diagnosis of intracerebral hemorrhage at baseline:*				
No	1 (ref. cat.)		1 (ref. cat.)	
Yes	2.66 (1.01–7.0)	0.048	2.82 (1.06–7.53)	**0.04**
*HAS-BLED score:*				
1-point increase	1.16 (0.86–1.55)	0.3	–	–

*OR* odds ratio, *CI* confidence interval, *adj.* adjusted, *ref. cat.* reference category

The considered variables were: age (at 5-year increase), HAS-BLED, and intracerebral hemorrhage as the cause leading to surgery.

The analysis showed that only intracerebral hemorrhage, as the cause of surgery, independently leads to an increased risk of postoperative hemorrhage in the surgical field (OR 2.82 [1.06–7.53], *p* = 0.04).

### Multivariate analysis for risk of post-operative thromboembolic events

Multiple logistic regression was also performed to analyze the correlation between the use of antiplatelets/anticoagulants before surgery and the development of postoperative thromboembolic events (Table 6).

**Table 6:** Multivariate analyses evaluating the association between anticoagulant-anti-PLT therapy status and post-surgical onset of thrombotic events, adjusting for selected potential confounders

	Thrombotic events (*n* = 38)		
	%	Adj. OR (95% CI)	p
Anticoagulant-anti-PLT therapy			
- no	18.6	1 (ref. cat.)	
- yes	41.7	1.69 (0.58–4.91)	0.3
*Age:*			
5-year increase	–	0.92 (0.78–1.07)	0.3
*Diagnosis of ischemia at baseline:*			
- no	18.5	1 (ref. cat.)	
- yes	48.7	3.49 (1.47–8.28)	**0.005**
*CHA2DS2-VASc score:*			
- 1-point increase	–	1.27 (0.93–1.74)	0.13

*OR* odds ratio, *CI* confidence interval, *adj.* adjusted, *ref. cat.* reference category

The variables considered were: age (5-year increase), CHA_2_DS_2_-Vasc, and ischemic stroke as possible causes leading to the intervention, but only this last one appeared significantly associated with an increased risk of postoperative thromboembolic events (OR 3.49 [1.47–8.28], *p* = 0.005).

### Multivariate analysis for risk of 1 and 6 month – post-operative unfavorable (1–3) GOS

A multiple logistic regression model was performed to analyze the correlation between the use of antiplatelet or anticoagulant medications before surgery and the development of 1 and 6 month – post-operative unfavorable (1–3) GOS (Table 7).

**Table 7:** Multivariate analyses evaluating the association between anticoagulant-anti-PLT therapy status and (a) 1-month unfavourable GOS, (b) 6-month unfavourable GOS, adjusting for selected potential confounders

		1-month GOS 1–3			6-month GOS 1–3	
	%	Adj. OR (95% CI)	p	%	Adj. OR (95% CI)	p
Anticoagulant-anti-PLT therapy						
- no	82.8	1 (ref. cat.)		69.6	1 (ref. cat.)	
- yes	100*	–	–	92.1	2.10 (0.39–11.3)	0.4
*Age:*						
5-year increase	–	1.14 (0.92–1.42)	0.2	–	1.08 (0.90–1.29)	0.4
*CCI:*						
- 1-point increase	–	1.20 (0.78–1.85)	0.4	–	1.11 (0.81–1.52)	0.5
*HAS-BLED score*:						
- 1-point increase	–	1.51 (0.0.57–3.98)	0.4	–	1.72 (0.85–3.48)	0.13
*CHA2DS2-VASc score:*						
- 1-point increase	–	1.12 (0.54–2.32)	0.8	–	0.79 (0.49–1.28)	0.3
*Pre-surgical GCS:*						
- 1-point increase	–	0.81 (0.67–0.97)	**0.025**	–	0.84 (0.73–0.98)	**0.022**

*OR* odds ratio, *CI* confidence interval, *adj.* adjusted, *ref. cat.* reference category, *CCI* Charlson Comorbidity Index, *GOS* Glasgow Outcome Scale, *GCS* Glasgow Coma Scale

^*^ In the model predicting 1-month GOS, the assumption of anticoagulant-anti-PLT therapy perfectly predicts the likelihood of the outcome, thus the covariate was dropped from the model. The raw % shows the proportion of patients with the outcome in each category of exposed and unexposed group (PLT therapy yes vs. no)

We considered age (5-year increase), CCI (1-point increase), HAS BLED score (1-point increase), CHAD2DS2-VASc score (1-point increase) and pre-surgical GCS (1-point increase) as possible variables associated with outcome, but only the last one showed a statistically significant correlation with a reduced risk for unfavorable GOS at 1 and 6 months OR = 0.81 (CI = 0.67–0.97, *p* = 0.025).

## Discussion

The evaluation of the risk associated with a previous therapy with antithrombotic/anticoagulant therapy in the context of decompressive craniectomy presents a complex interplay of potential benefits and risks. This study analyzed the influence of this therapy on postoperative hemorrhagic and thromboembolic events, alongside the overall patient outcomes at one and six months.

*Our findings suggest that a systematic discontinuation of antithrombotic therapy before decompressive craniectomy may not be necessary in all patients, and early resumption should be strongly considered to mitigate thromboembolic risk.*

### Antithrombotic therapy and postoperative hemorrhagic events

Surgeons are usually very concerned about the risk of hemorrhagic complications during and after surgery in patients taking anticoagulant or antithrombotic medications. The complexity of this issue is demonstrated by the absence of univocal data. On one hand, our data and recent evidence [5, 7] suggest that the use of these drugs was not associated with an increased risk of postoperative hemorrhagic events in patients undergoing DC; on the other hand data by Schuss et al. [8] showed that antiplatelet therapy significantly increased this risk.

In the present study, while a numerical increase in hemorrhagic complications was observed in the antithrombotic group (20.0% vs. 11.3%), this did not reach statistical significance. The confidence interval suggests that a larger cohort may be needed to fully determine whether a clinically meaningful difference exists.

Neither at the univariate nor multivariate analysis, statistically significant difference was found in postoperative bleeding incidence.

Indeed, a systematic review by Angelini et al. [5] showed no significant increase in rebleeding incidents among patients on antiplatelet or anticoagulant therapies compared to those not on such treatments​.

Han et al. [7] reported that preoperative antiplatelet therapy was not associated with a higher incidence of hemorrhagic complications in patients with traumatic brain injury (TBI) undergoing DC. Therefore, antiplatelet therapy should not delay time of surgical intervention.

Song et al. [11] conducted a study with similar findings, emphasizing that preoperative antiplatelet therapy did not have a statistically significant increase in rebleeding after treatment of intracerebral hemorrhage.

On the other hand, Schuss et al. [8] found that antiplatelet therapy significantly increased the risk of postoperative bleeding complications in stroke patients undergoing DC, but not intravenous thrombolytic therapy. They hypothesized ASA might influence pharmacokinetics and might enable possible drug interactions.

In our study, the intracerebral hemorrhage as an indication for surgery was the only factor negatively influencing the risk of postoperative hemorrhage (OR = 2.82 [1.06–7.53], p 0.04).

This finding underscores the importance of individualized patient assessments and suggests that blanket discontinuation of antithrombotic therapy may not be adequate. The decision to continue or interrupt the antithrombotic or anticoagulant therapy should be tailored, considering the type of surgery and the patient's overall health status. Indeed, A systematic interruption of anticoagulant and antiplatelet drugs or a resumption long after surgery may be unnecessary or even harmful to the patient. Moreover, it may not influence the risk of hemorrhagic events, which could occur regardless of the intake of these drugs. However, our findings showing a non-significant trends in hemorrhagic complications, should be always weighed up on the limitations of the study. Due to the limited sample size, we were unable to stratify results between anticoagulant and antiplatelet groups. Indeed, both types of drugs increase the risk of bleeding, but anticoagulants typically pose a higher risk of major bleeding events compared to antiplatelet drugs. This could represent a potential reason for non-significant trends in hemorrhagic complications, in addition to the patients’ heterogeneity.

### Antithrombotic-anticoagulant therapy and thromboembolic events

On the other hand, data showed 15.3% of strokes and 3.5% of acute myocardial infarction, which are higher and statistically significant (*p* < 0.001) in the AAPT group (27.7% vs 9.3% and 10.6% vs 0.0%). Therefore, ischemic complications are more frequent than hemorrhagic ones in general population, and the risk is particularly higher in the AAPT group. However, in multivariate analysis, this data is not confirmed, and the only factor that increases the risk is stroke as a cause of intervention. Indeed, if we evaluate the mean CHAD2DS2-VASc score, it is noted that for the AAPT group, it is 4.0 (vs 1.0 in the NT population), which represents a moderate risk for thromboembolic events. Therefore, in patients undergoing decompressive craniectomy for stroke, a careful assessment of thromboembolic risk factors should be performed, including the evaluation of the CHAD2DS2-VASc score, and early initiation of antiplatelet or anticoagulant therapy should be considered in moderate/high risk categories.

**Fig. 1:**
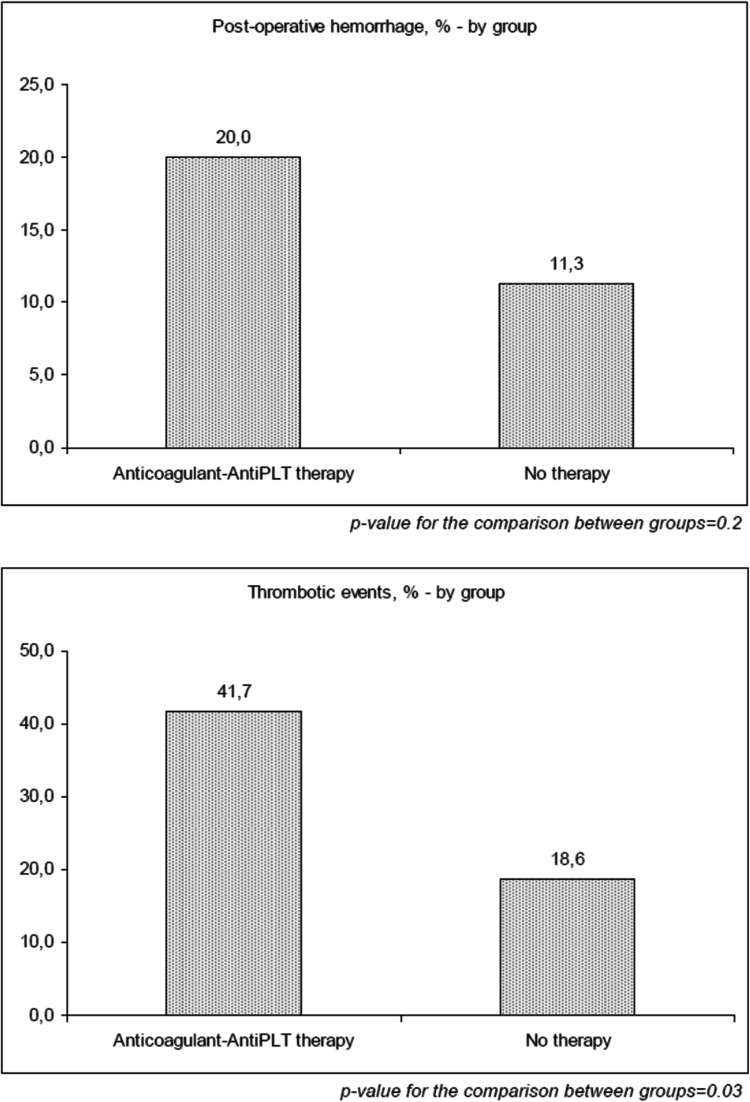
Frequency of post-operative hemorrhage (above) and thrombotic events (below), by treatment group

If it has been observed that the hemorrhagic risk does not seem to be increased by the use of these drugs, our data show that patients who used them before surgery have a moderate risk for thromboembolic events (see Fig. 1). For this reason, in these patients, discontinuation may not always be necessary, but at least an early resumption after surgery would be highly recommended.

### Antithrombotic-anticoagulant therapy and outcomes at 1 and 6 months

Our results confirmed the well-known [5, 6, 15] high % of morbidity for patients undergoing DC with 88.5% of patients with death or severe disability at 1-month follow-up and 76.9% at 6-month follow-up. Indeed, generally 1 patient out of 4 presents a minor disability at 6-month follow-up.

Univariate analysis showed a statistically significant difference for patients in the AAPT group with worse clinical outcomes (see Table 4).

However, the multivariate analysis did not confirm these data and the only factor reducing the risk of unfavorable outcome (GOS 1–3) at 1- and 6-month follow-up is the 1 point-increase of pre-surgical GCS (OR = 0.81, CI = 0.67–0.97, *p* = 0.025 and OR = 0.84, CI = 0.73–098, *p* = ns). The pre surgical GCS is also reported [16] to be the strongest predictor of successful extubation compared to many other factors including comorbid disease, vital signs, and respiratory function. Dobran et al. [17] in their study about prognostic factors and long-term follow-up in patients undergoing DC, reported admission GCS > 8 was significantly associated with six months good outcome.

Angelini et al. [5] observed that six-month neurological outcomes were similar between patients on antiplatelet/anticoagulant (AP/AC) therapy and those not receiving these medications.

Kinoshita et al. [18] found that while decompressive craniectomy performed for the evacuation of intracranial hemorrhagic lesions was associated with worse outcomes in elderly TBI patients, there were no discernible differences in the outcomes between patients treated with antithrombotics and those not treated with them.

Although a lack of correlation at multivariate analysis was found between the use of these drugs and the clinical outcome, we should consider patients taking these medications as very complex and fragile for their comorbidities. In these patients the baseline therapy should be respected, and this could be another reason not to adopt a long-time suspension time for anticoagulants or antiplatelets.

## Limitations

The number of patients enrolled, even if higher than similar studies, remains limited. Moreover, different type of baseline diagnosis (trauma, stroke or intracerebral hemorrhage) were considered together. Indeed, this could influence the final clinical outcome or complications (DC in trauma or stroke is usually a last line of therapy and brain injury could be already very diffuse; intracerebral hemorrhage has been shown to be the only factor increasing the hemorrhagic risk).

Due to the limited number of patients a sub-analysis per type of medication (ASA, NOAC, Vitamin k antagonist, ecc ecc) was not possible. A sub-analysis of the type of antithrombotic therapy (e.g., aspirin, NOACs, or dual therapy) and specific patient characteristics (e.g., age, baseline diagnosis) would provide more actionable insights.

Due to the lack of practical guidelines about the management of these kind of drugs in patients undergoing DC, we did not apply any standard protocol. We were only able to collect time of suspension and resumption of the drugs.

To minimize this bias, we could employ several strategies, such as blinding outcome assessors to the treatment groups to reduce assessment bias. Evaluation of outcome, hemorrhagic, or thromboembolic events could be performed by a blinded assessor. Another strategy to reduce bias could be standardizing treatment protocols across all participating centers. This included uniform guidelines for the suspension and resumption of antithrombotic drugs and postoperative care.

While sensitivity analyses were not conducted, the multivariate models were built using clinically relevant variables, and overfitting was minimized.

Regarding the CHA2DS2-VASc and HAS-BLED scores, at multivariate analysis there was no significant influence on thromboembolic or hemorrhagic risk. For this reason, the identification of a proper threshold for clinical decision guidance was not possible. We believe that this identification could be very useful and we plan to keep on collecting more data about these scores in order to try to identify possible threshold values.

Finally, while outcome assessors were not blinded to the treatment groups, objective criteria were used for defining haemorrhagic and thromboembolic complications to minimise bias.

## Conclusions

Our data suggest that pretreatment with antithrombotic or anticoagulant medication does not necessarily increases the risk of postoperative hemorrhagic events in DC patients. However, the type of surgery and the patient's baseline characteristics play crucial roles in assessing this risk, highlighting the importance of individualized patient assessments. While there is an initial association between antithrombotic therapy and an increased risk of postoperative thromboembolic events, further analysis suggests that surgery-related factors, such as undergoing the procedure due to ischemic stroke, contribute more significantly to this risk. Optimizing perioperative management of antithrombotic therapy is essential to mitigate these risks, with strategies like bridging anticoagulation offering potential solutions. Scores like CHAD2DS2-VASc could be helpful to stratify the patient risk.

## Data Availability

No datasets were generated or analysed during the current study.
